# Supplementary material for: Long-Range PCR-Based NGS Applications to Diagnose Mendelian Retinal Diseases
Source: Int J Mol Sci. 2021 Feb 3;22(4):1508. doi: 10.3390/ijms22041508 (PMC7913364; doi:10.3390/ijms22041508)
Supplement: Supplementary file 1 [file ijms-22-01508-s001.zip › Supplementary_Files/Supplementary_Text_S1_LR_sequencing_protocol_edited.docx]

**Long-range PCR library prep protocol**

1. **gDNA dilution**
   1. Normalize all gDNA templates to the same concentration (10 ng/µl) in IDTE buffer (TE buffer pH 8.0) or ddH_2_O
   2. Check DNA concentration with NanoDrop
2. **Long-range PCR**
   1. Complete the following table listing all PCR reactions to be done with the corresponding templates

- 1. Insert the total number of reactions in the following table (calculate a 10% excess for pipetting errors):

- 1. Thaw 10X Buffer, 10X S-solution, dNTPs, region-specific primers, and the selected gDNA templates at RT
  2. Prepare the universal MM without primers according to the table above
  3. Mix well by pipetting up and down
  4. Prepare region-specific master mixes from the universal MM, if necessary:
     1. Complete the primer-specific MM table according to the number of reactions needed (calculate a 10% excess for pipetting errors) for each MM needed

- 1. From the primerless MM, create primer-specific Master Mixes according to the mixes above
  2. Mix well each primer-specific MM by pipetting up and down
  3. For small numbers of reactions, prepare an appropriate amount of 0.2 ml PCR tubes and label them
  4. Otherwise, plan how to distribute the reactions in a PCR plate:

- 1. Distribute 25 µl of primer-specific MM to the corresponding tubes or wells
  2. Add 5 µl of the template gDNA dilutions (50 ng of DNA in total)
  3. Mix by pipetting up and down
  4. Place the PCR tubes/plate on the thermal cycler with the **Long-Range 2-Step PCR** program:

| Stage | Temperature (Lid: 105°C) | Time | Number of cycles |
| --- | --- | --- | --- |
| Initial denaturation | 94°C | 2 min | 1 |
| Denaturation, annealing and elongation | 98°C | 10 s | 35 |
|  | 68°C | 12 min |  |
| Final elongation | 72°C | 10 min | 1 |
| Hold | 4°C | ∞ |  |

1. **Electrophoresis**
   1. Preparation:
      1. Depending on how many PCR reactions there are, decide on a particular gel size with corresponding comb
      2. Prepare the chosen gel chamber by sealing the sides with tape so that the liquid gel solution is confined to the chamber
   2. Prepare a 0.6% agarose gel for the chosen quantity
      1. E.g. for a 100-ml gel pour the following mix into a 250-ml Erlenmeyer flask:

| Reagent | Quantity |
| --- | --- |
| Agarose powder | 0.6 g |
| 1X TAE buffer | 100 ml |

- 1. Heat the mix in a microwave oven until it starts boiling and the solution is completely clear
  2. Wait for the mixture to cool down and/or speed up the cooling process by swirling the solution in the Erlenmeyer flask under cold running tap water until it reaches a temperature of approximately 60-70˚C
  3. Transfer the solution into an EthBr-dedicated Erlenmeyer
  4. Add an appropriate amount of EthBr reagent
     1. E.g. for a 100-ml gel add 5 µl
  5. Mix gently by swirling the solution
  6. Carefully pour the gel solution onto the previously taped gel chamber
  7. Ensure no bubbles remain
  8. Add the appropriate comb(s)
  9. Let the gel cool down and solidify for approximately 30 minutes at RT
  10. Put the gel chamber in the cold room (or in the fridge) for approximately one hour
      1. Before taking the gel out of the cold room, prepare the samples to be loaded
  11. Thaw the GeneRuler High Range DNA Ladder and its loading dye at RT
  12. Prepare the following mixes to be loaded:

| Sample | Sample amount | Loading Dye | ddH_2_O |
| --- | --- | --- | --- |
| Ladder | 0.5 µl | 1 µl | 4.5 µl |
| PCR reaction | 2.5 µl | 1 µl | 2.5 µl |

- 1. Move the gel from the cold room (or fridge) into the electrophoresis chamber
  2. Make sure the gel is covered with TAE buffer
  3. Slowly and carefully remove the comb(s), making sure not to damage the wells
  4. Load 6 µl of ladder mix into the correct well(s)
  5. Load 6 µl of PCR reaction mix into the correct well(s)
  6. Run the gel at 60V for 2-3 hours
  7. Carefully place the gel onto the imager and image it

1. **QuBit concentration measurement**
2. Preparation:
3. Equilibrate Qubit dsDNA HS Buffer at RT for 30 min
4. Thaw enough Qubit dsDNA HS Reagent at RT and keep in a dark place
5. Prepare enough Qubit tubes and label them, including 2 for the Standards
6. Prepare enough Working Solution, including 10% excess for pipetting errors (make sure to include 2 standards in the calculation):
7. Complete the following table:

1. Mix thoroughly by pipetting up and down
2. Add 190 µl of Working solution into the tubes for the Standards
3. Add 199 µl of Working solution into the tubes for the samples
4. Add 10 µl of Standards into the corresponding tubes
5. Add 1 µl of sample into the corresponding tubes
6. Vortex for 3 s
7. Allow all tubes to incubate at RT for 2 min
8. Turn the Qubit fluorometer on
9. Select the dsDNA High Sensitivity measurement
10. Read the Standards
11. Measure the samples and note the results into the Excel sheet:

1. Decide how to pool the PCRs together and how the libraries will be composed and fill the following table
   1. Pools can contain all amplicons belonging to the same gDNA template
   2. It is also possible to pool together non-overlapping amplicons from different gDNA template to reduce the number of libraries to be prepared
      1. E.g. amplicons for different loci

1. Pool all PCR products of the pool together (ideally for at least 130 µl total volume)
2. **DNA shearing**
3. Preparation:
4. If frozen, thaw the PCR pools
5. Turn Covaris laptop and ultrasonicator on
6. Fill Covaris well with AFA water to the right level
7. Place the appropriate Holder XTU
8. Depending on which sequencing reagents will be used, decide on a mean target size for the fragments
9. E.g. to optimise the use of a 500-cycles kit, the ideal mean insert size is about 500-600 bp long
10. For a 300-cycles kit, the ideal mean insert size is about 300-350 bp long
11. Pipette 130 µl of PCR pool into a 130 µl Covaris AFA microTube
12. Choose an appropriate shearing protocol for the library target size

| Target size (for 130 µl tubes) | Peak incident Power | Duty factor | | Cycles per burst | Treatment Time |
| --- | --- | --- | --- | --- | --- |
| 500 | 50 W | 10% | 200 | | 50 s |
| 400 | 50 W | 10% | 200 | | 70 s |
| 300 | 50 W | 20% | 200 | | 65 s |

1. After shearing, transfer 130 µl of sheared pool into a fresh 1.5-ml tube
2. **Library Quantification and size distribution check with Bioanalyzer**
3. Preparation:
4. Check if DNA High Sensitivity Gel-Dye Mix aliquot is available
   - - - If not, prepare it as described in the Agilent DNA High Sensitivity protocol
5. Allow the gel-dye mix to equilibrate at room temperature for 30 min
6. Allow the marker and ladder to equilibrate at room temperature for 30 min
7. Assemble priming station
   - - - Make sure the clip lever is at the lowest position
8. Put a new DNA High Sensitivity chip on the priming station
9. Make sure the plunger is positioned at 1 ml
10. Pipette 9 µl of gel-dye mix in the well marked **G**
11. Close the priming station
12. Press the plunger until it is held by the clip
13. Wait for exactly 60 s, then release the clip
14. Wait for 5 s and slowly pull the plunger back up at 1 ml
15. Open the priming station
16. Pipette 9 µl of gel-dye mix in the other 2 wells marked with G
17. Pipette 5 µl of marker (green cap) in all 12 sample wells and the ladder well
18. Do not leave any wells empty
19. Pipette 1 µl of DNA ladder (yellow cap) in the well marked with a ladder (bottom right)
20. Pipette 1 µl of sample in the sample wells
21. Pipette 1 µl of water in the unused wells
22. Put the chip horizontally in the chip vortexer and run for 1 min at 2400 rpm
23. Run the chip on the Bioanalyzer within 5 min
24. Fill the table with the mean size of each sample pool

At this point, the sample pools are ready for the NGS library preparation procedure. For this step, different library prep kits are available (e.g. KAPA Hyper, Thruplex DNA-seq, Illumina TruSeq Nano DNA kit). For the TruSeq Nano DNA kit:

1. **Library prep – End Repair**
2. Preparation:
   1. Thaw sheared sample pools at RT
   2. Equilibrate SPB at RT
   3. Equilibrate RSB at RT
   4. Thaw Illumina ERP2 (or ERP3) at RT and then place on ice
   5. Reserve a Thermocycler and make sure the ERP program is saved
3. Distribute 60 µl of sheared sample pools into different 0.2 ml PCR tubes and label them
4. Add 40 µl of ERP2 to each tube
5. Place the PCR tubes/plate on the thermal cycler with the **Illumina TruSeq Nano ERP** program:

| Stage | Temperature (Lid: 100°C) | Time |
| --- | --- | --- |
| End repair | 30°C | 30 min |
| Hold | 4°C | ∞ |

1. Heated lid: 100°C
2. Total volume: 100 µl
3. **Library prep – Remove large DNA fragments**
4. Preparation:
   1. Prepare an appropriate amount of 80% EtOH (600 µl per 1.5-ml tube) with a 10% excess for pipetting errors
   2. Vortex SPB until well-dispersed
5. Dilute SPB with ddH_2_O according to the following formula (this formula is for a target size of 350 bp):

1. Vortex again
2. Transfer each ERP reactions products (100 µl) to a fresh 1.5-ml tube
3. Add 160 µl of diluted SPB to each ERP sample from step b.
4. Vortex and centrifuge briefly
5. Incubate at RT for 5 min
6. Put the tubes onto the appropriate magnet
7. Wait for the solution to clear up
8. After 30 s, rotate the tubes by 90° and repeat 3 more times until they are back to the original position
9. Let stand for an additional 2 min
10. Transfer 250 µl of supernatant to a fresh 1.5-ml tube labeled accordingly
11. Discard remaining diluted SPB
12. **Library prep – Remove small DNA fragments**
13. Preparation:
    1. Prepare an appropriate amount of 80% EtOH (600 µl per 1.5-ml tube) with a 10% excess for pipetting errors
    2. Vortex SPB until well-dispersed
14. Add 30 µl undiluted SPB to each tube
15. Vortex and centrifuge briefly
16. Incubate at RT for at least 5 min for maximum recovery
17. Put the tubes onto the appropriate magnet
18. Wait for the solution to clear up
19. While keeping the tubes on the magnet, aspirate and discard the supernatant
    1. Leave 5 µl behind to avoid the beads to come with the supernatant
20. Add 300 µl of 80% EtOH to each tube
21. After 30 s, rotate the tubes by 90° and repeat 3 more times until they are back to the original position
22. Remove the supernatant without disturbing the beads
23. Repeat steps h-j
24. Remove residual EtOH with a 20 µl pipette tip
25. Leave the tubes/wells open to dry the residual EtOH for approximately 3.5 min (do not overdry)
26. Remove the tubes from the magnet
27. Add 22 µl of RSB
28. Mix by pipetting up and down
29. Vortex and centrifuge briefly
30. Incubate at RT for 4 min
31. Place the plate/tubes onto the magnet again
32. Wait for the solution to clear up
33. After 30 s, rotate the tubes by 90° and repeat 3 more times until they are back to the original position
34. Transfer 17.5 µl of the eluate to a new PCR tube


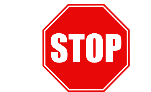


1. **Library prep – A-Tailing**
2. Preparation:
3. Equilibrate RSB at RT for 30 min
4. Thaw ATL (or ATL2) at RT
5. Reserve a Thermocycler and make sure the ATAIL70 program is saved
6. Centrifuge ATL (or ATL2) briefly
7. Add 12.5 µl of ATL (or ATL2) to each PCR tube
8. Mix by pipetting up and down
9. Centrifuge briefly
10. Place the PCR tubes/plate on the thermal cycler with the **Illumina TruSeq Nano ATAIL70** program:

| Stage | Temperature (Lid: 100°C) | Time |
| --- | --- | --- |
| A-Tailing | 37°C | 30 min |
| Stop reaction | 70°C | 5 min |
| Cool down | 4°C | 5 min |
| Hold | 4°C | ∞ |

1. Proceed immediately to adapter ligation
2. **Library prep – Adapter ligation**
3. Preparation:
   1. Equilibrate RSB at RT for 30 min
   2. Equilibrate SPB at RT for 30 min
   3. Thaw STL at RT
   4. Thaw selected DNA adapter aliquots at RT
   5. Reserve a thermocycler and make sure the LIG program is saved
4. Briefly centrifuge the adapters
5. Briefly centrifuge the samples
6. Remove LIG2 from the freezer and place on ice
7. Add the following reagents in the order listed to each tube:

| Reagent | Volume |
| --- | --- |
| RSB | 2.5 µl |
| LIG2 | 2.5 µl |
| DNA adapters | 2.5 µl |

1. Mix by pipetting up and down
2. Centrifuge briefly
3. Place the PCR tubes/plate on the thermal cycler with the **Illumina TruSeq Nano LIG** program:

| Stage | Temperature (Lid: 100°C) | Time |
| --- | --- | --- |
| Ligation | 30°C | 10 min |
| Hold | 4°C | ∞ |

- 1. Volume: 38 µl

1. Centrifuge briefly
2. Immediately add 5 µl of STL to each tube to stop the reaction
3. Transfer 42.5 µl from the PCR tube to a fresh 1.5-ml tube
4. Vortex and centrifuge briefly
5. **Library prep – Clean up ligated fragments**
6. Preparation:
   1. Prepare an appropriate amount of 80% EtOH (600 µl per 1.5-ml tube) with a 10% excess for pipetting errors
   2. Vortex SPB until well-dispersed
7. Add 42.5 µl undiluted SPB to each tube
8. Vortex and centrifuge briefly
9. Incubate at RT for at least 5 min for maximum recovery
10. Put the tubes onto the appropriate magnet
11. Wait for the solution to clear up
12. While keeping the tubes on the magnet, aspirate and discard the supernatant
    1. Leave 5 µl behind to avoid the beads to come with the supernatant
13. Add 300 µl of 80% EtOH to each tube
14. After 30 s, rotate the tubes by 90° and repeat 3 more times until they are back to the original position
15. Remove the supernatant without disturbing the beads
16. Repeat steps h-j
17. Remove residual EtOH with a 20 µl pipette tip
18. Let the tubes/wells open to dry the residual EtOH for about 3.5 min (do not overdry)
19. Remove the tubes from the magnet
20. Add 54 µl of RSB
21. Mix by pipetting up and down
22. Vortex and centrifuge briefly
23. Incubate at RT for 5 min
24. Place the tubes onto the magnet again
25. After 30 s, rotate the tubes by 90° and repeat 3 more times until they are back to the original position
26. Transfer 50 µl of the eluate to a new 1.5-ml tube
27. Add 50 µl undiluted SPB to each tube
28. Vortex and centrifuge briefly
29. Incubate at RT for at least 5 min for maximum recovery
30. Put the tubes onto the appropriate magnet
31. Wait for the solution to clear up
32. While keeping the tubes on the magnet, aspirate and discard the supernatant
    1. Leave 5 µl behind to avoid the beads to come with the supernatant
33. Add 300 µl of 80% EtOH to each tube
34. After 30 s, rotate the tubes by 90° and repeat 3 more times until they are back to the original position
35. Remove the supernatant without disturbing the beads
36. Repeat steps bb-dd
37. Remove residual EtOH with a 20 µl pipette tip
38. Leave the tubes open to dry the residual EtOH for approximately 3.5 min (do not overdry)
39. Remove the tubes from the magnet
40. Add 29 µl of RSB
41. Mix by pipetting up and down
42. Vortex and centrifuge briefly
43. Incubate at RT for 5 min
44. Place the tubes onto the magnet again
45. After 30 s, rotate the tubes by 90° and repeat 3 more times until they are back to the original position
46. Transfer 25 µl of the eluate to a new PCR tube


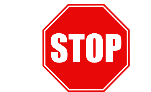


1. **Library prep – Library amplification**
2. Preparation:
3. Equilibrate PPC at RT. Do not vortex
4. Thaw EPM on ice
5. If frozen, thaw the samples with beads at RT
6. Make sure the thermal cycler is reserved and has the amplification program saved
7. If amplifying more than one pooled library, complete reaction mix table on b.
8. If continuing with purification right away, equilibrate SPB at RT for 30 min
9. Prepare PCR master mix, if necessary:

1. If a PCR master mix is prepared, add 25 µl to each tube
2. Otherwise, add 5 µl of PPC and 20 µl of EPM to each tube
3. Mix by pipetting up and down
4. Briefly centrifuge
5. Place the PCR tubes onto the thermal cycler with the **Illumina TruSeq Nano PCR** program:

| Stage | Temperature (Lid: 100°C) | Time | Number of cycles |
| --- | --- | --- | --- |
| Polymerase activation | 95°C | 3 min | 1 |
| Amplification | 98°C | 20 s | 12 |
|  | 60°C | 15 s |  |
|  | 72°C | 30 s |  |
| Final Extension | 72°C | 5 min | 1 |
| Hold | 4°C | ∞ | 1 |

1. Volume: 50 µl
2. **Post-amplification PCR purification**
3. Preparation:
4. Equilibrate SPB at RT for 30 min
5. Equilibrate RSB at RT for 30 min
6. Prepare enough fresh 80% ethanol (600 µl per tube)
7. Vortex the beads until solution is homogeneous
8. Transfer the PCR volume (50 µl) to a fresh 1.5-ml tube
9. After vortexing, add 50 µl (1X volume) of SPB to the amplified libraries
10. Pipette up and down to mix
11. Vortex briefly
12. Incubate at RT for 5 min
13. Put tubes on magnetic stand
14. After 30 s, rotate the tubes by 90° and repeat 3 more times until they are back to the original position
15. With the tube on the magnetic stand, discard the supernatant without touching the beads
    1. Leave 5 µl behind to avoid the beads to come with the supernatant
16. Add 300 µl of 80% ethanol to each tube
17. After 30 s, rotate the tubes by 90° and repeat 3 more times until they are back to the original position
18. Aspirate and discard the supernatant
19. Add 300 µl of 80% ethanol to each tube
20. After 30 s, rotate the tubes by 90° and repeat 3 more times until they are back to the original position
21. Aspirate and discard the supernatant
22. With the tube on the magnetic stand, discard the residual ethanol without touching the beads with a 20 µl tip
23. Dry the beads at RT for 3.5 min
24. Attention: Do not over dry the pellets (over dried pellets appear cracked)
25. Remove tubes from magnet
26. Elute the DNA by re-suspending the beads it in 35 µl of Resuspension Buffer
27. Pipette up and down to mix
28. Centrifuge very briefly
29. Incubate at RT for 5 min
30. Put on magnetic stand
31. After 30 s, rotate the tubes by 90° and repeat 3 more times until they are back to the original position
32. With the tube on the magnetic stand, transfer 31 µl of supernatant into a new tube
33. If not used immediately, the purified library can be stored at -20°C for up to a week or at 4°C overnight


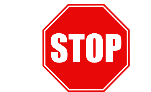


1. **Validate and quantify library**
2. Preparation:
3. Check if High Sensitivity Gel-Dye Mix aliquot is available
   - - - If not, prepare it as described in the Agilent DNA High Sensitivity Kit protocol
4. Allow the gel-dye mix to equilibrate at room temperature for 30 min
5. Allow the marker and ladder to equilibrate at room temperature for 30 min
6. Assemble priming station
   - - - Make sure the clip lever is at the lowest position
7. Put a new DNA High Sensitivity chip on the priming station
8. Make sure the plunger is positioned at 1 ml
9. Pipette 9 µl of gel-dye mix in the well marked **G**
10. Close the priming station
11. Press the plunger until it is held by the clip
12. Wait for exactly 60 s, then release the clip
13. Wait for 5 s and slowly pull the plunger back up at 1 ml
14. Open the priming station
15. Pipette 9 µl of gel-dye mix in the other 3 wells marked with G
16. Pipette 5 µl of marker (green cap) in all 11 sample wells and the ladder well
17. Do not leave any wells empty
18. Pipette 1 µl of DNA ladder (yellow cap) in the well marked with a ladder (position 12)
19. Pipette 1 µl of sample in the sample wells (in duplicate if possible)
20. Pipette 1 µl of water in the not used wells
21. Put the chip horizontally in the chip vortexer and vortex for 1 min at 2400 rpm
22. Run the chip on the Bioanalyzer within 5 min
23. Samples can be stored at -20°C
24. Analyze the size of the libraries and make sure that the mean size increased
    1. Adapters that have been ligated should increase the size of the library
25. **QuBit library concentration measurement**
26. Preparation:
27. Equilibrate Qubit dsDNA HS Buffer at Rt for 30 min
28. Thaw enough Qubit dsDNA HS Reagent at RT and keep in dark
29. Prepare enough Qubit tubes and label them, including 2 for the Standards
30. Each library will be measured twice: undiluted and its 1:10 dilution
31. Prepare enough Working Solution, including 10% excess for pipetting errors:
32. Complete the following table (count each library twice and add the 2 standards)

1. Prepare the master mix accordingly
2. Mix thoroughly by pipetting up and down
3. Add 190 µl of Working solution into the tubes for the Standards
4. Add 199 µl of Working solution into the tubes for the samples
5. Add 10 µl of Standards into the corresponding tubes
6. Add 1 µl of sample into the corresponding tubes
7. Vortex for 3 s
8. Allow all tubes to incubate at RT for 2 min
9. Turn the Qubit fluorometer on
10. Select the dsDNA High Sensitivity measurement
11. Read the Standards
12. Measure the samples
13. Annotate the concentration results into the Excel sheet (columns G and H) and add the mean library size from the Bioanalyzer results (column J)

1. Complete the following Excel sheet to calculate molarity and how to dilute to a target 4 nM

1. Dilute each library according to column “Pool vol.” and “RSB”
2. Pool the 4nM libraries into a final 4nM sequencing library proportionally to the total target size of each pool:

1. **Loading library onto MiSeq**
   1. Preparation:
      1. Thaw 2N NaOH at RT
      2. Wash the MiSeq instrument, if necessary
      3. Thaw HT1 at RT
      4. Chill thawed HT1 on ice (or in the fridge)
      5. Prepare PhiX library, if necessary
      6. Equilibrate flow cell at RT for 1 hour
      7. Note the serial number of the reagent cartridge to be used
      8. Thaw reagent cartridge in water (check max. water level line on the side) for 1 hour
      9. Place reagent cartridge in fridge until ready to load library
      10. Thaw PR2 bottle in the fridge for 1 hour
   2. Prepare fresh 0.2N NaOH in a fresh 1.5-ml tube:
      1. Add 45 µl of ddH_2_O
      2. Add 5 µl of 2N NaOH
   3. In a new tube, add 5 µl of final 4 nM library (from step 16 ee)
   4. Add 5 µl of 0.2 N NaOH
   5. Vortex briefly
   6. Centrifuge briefly
   7. Incubate at RT for 5 min
   8. Add 990 µl of pre-chilled HT1 to the denatured library
      1. This results in 1 ml of 20 pM denatured library
      2. Place on ice
   9. If necessary, dilute to the desired loading concentration:

| Concentration | 8 pM | 10 pM | 12 pM | 15 pM | 20 pM |
| --- | --- | --- | --- | --- | --- |
| 20 pM library | 240 µl | 300 µl | 360 µl | 450 µl | 600 µl |
| Pre-chilled HT1 | 360 µl | 300 µl | 240 µl | 150 µl | 0 µl |

- - 1. Our experience so far tells us that a 12-13 pM library results in a good cluster density
  1. Place library ready to be loaded on ice
  2. Prepare sample sheet on the instrument with the reagent cartridge serial number information
  3. Once the instrument is ready to be used, start the sequencing protocol on it
  4. Wash the flow cell carefully to remove solution salts
     1. First spray it with ddH_2_O all over
     2. Use a lint-free paper tissue to dry it
     3. Secondly, spray the glass surface on both sides with ethanol, avoiding putting ethanol on the black plastic ports
     4. Dry and clean the glass surface with a lint-free paper tissue
     5. Once clean and dry, place on instrument according to instruction
  5. Follow the instruction from the instrument and load the PR2 bottle and an empty waste bottle
  6. Remove reagents cartridge from the fridge
  7. Invert at least 10 times to mix
  8. Pierce the cartridge well number 17 (highlighted in orange) with a 1000 µl tip
  9. Pipette 600 µl of the final denatured library into the well number 17
  10. Wait for the software for the prompt to load the reagent cartridge
      1. Remove the wash cartridge
      2. Insert the reagent cartridge **containing the library** until it cannot slide anymore
  11. Close the cartridge door
  12. Click next and check if the correct sample sheet will be loaded and if the sequencing details are correct
  13. Press next
      1. At this point the instrument will check its functionality and the fluidic systems
  14. Once the pre-run check is done, click on Start
  15. The run will start
  16. The cluster density info will appear after cycle 15 (roughly 2-3 hours)
  17. The Q30 statistics will appear after cycle 25 (roughly 3h 30 min)
