# Supplementary material for: Long-Range PCR-Based NGS Applications to Diagnose Mendelian Retinal Diseases
Source: Int J Mol Sci. 2021 Feb 3;22(4):1508. doi: 10.3390/ijms22041508 (PMC7913364; doi:10.3390/ijms22041508)
Supplement: Supplementary file 1 [file ijms-22-01508-s001.zip › Supplementary_Files/Supplementary_Figure_S2_legend_WES_spike-in.pdf]

**Figure S2. WES spike-in proof of concept.**

Screenshot from the Alamut Visual software showing alignment of different assays for the *ABCA4* (A), *RPGR* (B), and *PCARE* (C) loci. On top, the alignment resulting from a typical WES assay. In the middle, the results of the spike-in of IDT custom capture probes for *ABCA4* are shown (A). On the bottom, the long-range PCRs library spike-in results are illustrated.

A)

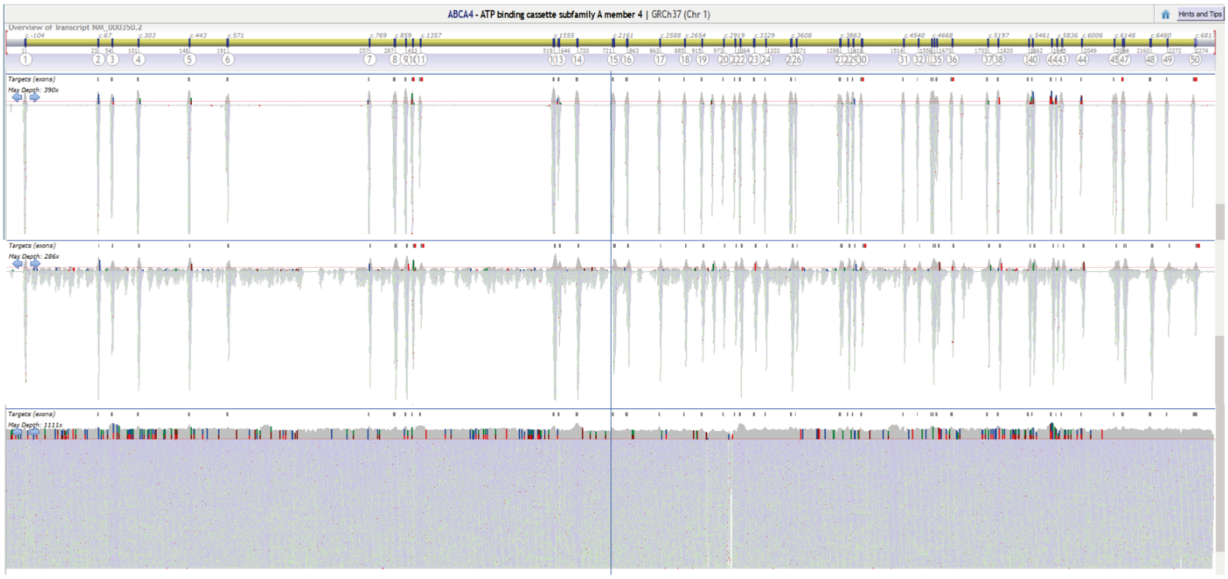
ABCA4 - ATP binding cassette subfamily A member 4 | GRCh37 (Chr 1)
Hints and Tips
Overview of Transcript NM_000350.2
Targets (exons)

B)

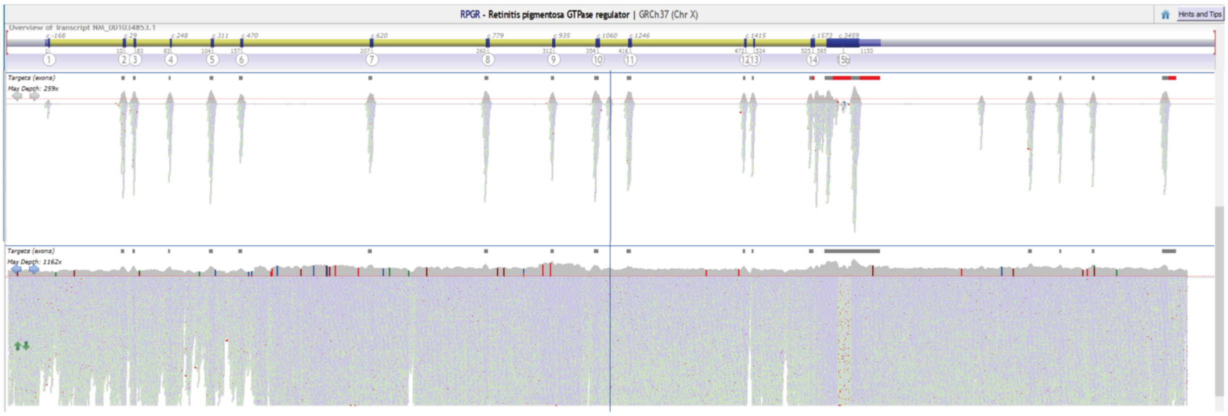
RPGR - Retinitis pigmentosa GTPase regulator | GRCh37 (Chr X)
Hints and Tips
Targets (exons)

C)

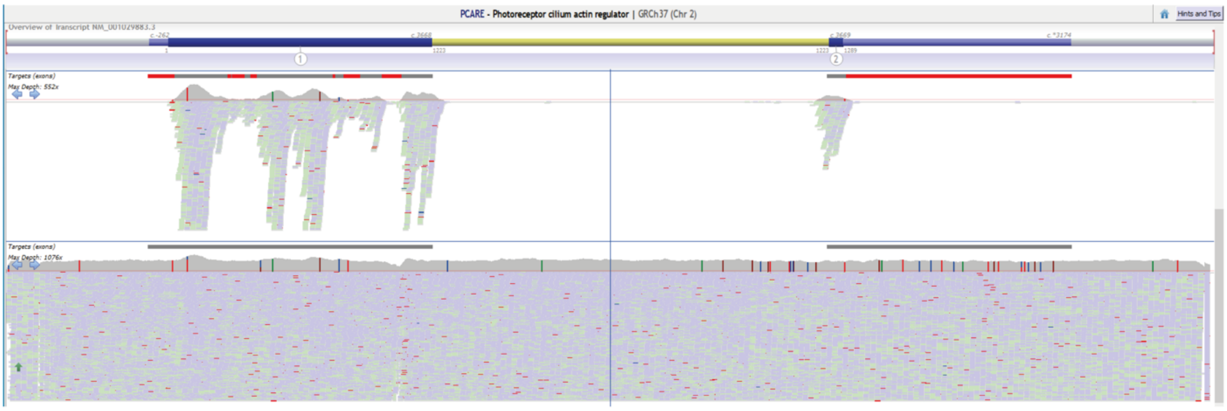
PCARE - Photoreceptor cilium actin regulator | GRCh37 (Chr 2)
Hints and Tips
Targets (exons)
